# Gap analysis between expectations and perceptions of pregnant women attending Prevention of Maternal to Child Transmission of HIV services in a private referral hospital in northern Tanzania: A cross-sectional descriptive study

**DOI:** 10.1371/journal.pone.0257771

**Published:** 2021-09-22

**Authors:** Lugata John, Nesister Odero, Jackson Nziku, Bernard Njau

**Affiliations:** 1 Kilimanjaro Christian Medical University Collage, Moshi, Tanzania; 2 Kilimanjaro Christian Medical Centre, Moshi, Tanzania; University of Mississippi Medical Center, UNITED STATES

## Abstract

**Objective:**

Pregnant women satisfaction with the Prevention of Mother-To-Child HIV Transmission services is an essential parameter in the determination of the quality of care and performance. This study aimed to measure the gap between pregnant women expectations of PMTCT services and perceptions of the actual PMTCT services and the relationship between their service gap scores and socio-demographic characteristics.

**Methods:**

A cross-sectional descriptive study design was conducted from August to September 2020 on a sample of 105 participants. A pre-tested SERVIQUAL questionnaire was used to collect data and paired sample t-test, independent one-sample t-test, and one–way ANOVA was used to compare mean service gap scores. A p-value of < 0.05 was considered statistically significant.

**Results:**

The overall mean gap score was (+ 0.31) indicating pregnant women perceived value of the quality of care of PMTCT services. The gap score in the 5 service dimensions was as follows: empathy (+0.49), tangibles (+0.43), assurance (+0.22), responsiveness (+0.20), and reliability (+0.19). Marital status (p-value 0.031) was the only social demographic characteristic associated with pregnant women service gap scores.

**Conclusion:**

Overall, pregnant women perceptions of PMTCT services provided in the RCH clinic at KCMC were meet. Marital status was associated with the overall pregnant women service gap scores and perceived quality of care with PMTCT services. Pregnant women who were married had small service gap scores compared to either divorced or widowed or cohabiting women.

## Introduction

In 2019, 60% of new HIV infection was reported among Women Of Reproductive Age (WRA; 15–49 years—an age group at high risk of HIV infection) in sub-Saharan Africa (SSA). Similarly, over 90% of new infections among children fewer than 15 years were in SSA, whereby Mother-To-Child Transmission (MTCT) contributes to over 90% of HIV infection in newborns [1]. Without antiretroviral treatment (ART), the likelihood of MTCT is 15% to 45% [1]. This risk however can be reduced to below 5% through Prevention Of Mother-To-Child Transmission (PMTCT) programmes [1].

Between 2010 and 2015, new HIV infection in WRA decreased by less than 2% and by 6% among adolescent and young women (15 to 24 years). In 2016, over 5.2 million WRA were infected with HIV globally, with 23 so-called high-priority for PMTCT countries, including the United Republic of Tanzania accounted for 87% of new HIV infections in children and 81% in adolescent girls and young women [1].

Pregnant women’ satisfaction with PMTCT services is a core determinant for the assessment of the quality of care [2]. However, in developing countries, including Tanzania, the quality of care of PMTCT remains a challenge, with variations existing between countries. For example pregnant women satisfaction varied from 92% in Tanzania [3], and 74.7% to 82.2% in Ethiopia [4, 5]. However, most studies focused on assessing the quality of PMTCT services by measuring pregnant women perception only, missing out on their expectations or the gap analysis between clients’ expectations and perceptions [3–5]. The Service Quality (SERVIQUAL) scale measures the gap between clients’ expectations and perceptions for measuring satisfaction and service quality [6], to improve quality of care [7].

In this study, the SERVQUAL model was adapted to measure the gap between pregnant women expectations of PMTCT services and perceptions of the actual PMTCT services and the relationship between their service gap scores and socio-demographic characteristics. The findings will identify areas of PMTCT services provided in the Reproductive and Child Health (RCH) clinic at the Kilimanjaro Christian Medical Centre (KCMC) private referral hospital in northern Tanzania that may need improvement.

## Methods

### Study area and design

A descriptive cross-sectional study design was used among pregnant women attending the RCH clinic at KCMC private referral hospital in Moshi Municipality of Kilimanjaro region. The study was conducted from August to September 2020.

Kilimanjaro is among 30 regions of Tanzania, geographically located in the Northeastern part of Tanzania, bordered to the north and east by Kenya, to the south by the Tanga region, to the west by Arusha region and the southwest by Manyara region. The region has seven administrative districts, namely Moshi Urban, Moshi Rural, Hai, Mwanga, Rombo, Same, and Siha.

Moshi municipality is located in Moshi Urban at the bottom of Mount Kilimanjaro, a tourist site with approximated 184,292 populations in the northern part of Tanzania [8].

KCMC is a faith-based private referral and teaching hospital with a 619-bed capacity, 1,000 health care providers, and an approximately 15.7 million catchment population. KCMC provides consultancy services to patients/ clients from the Kilimanjaro Region and other parts of northern Tanzania. Preventive services provided at KCMC include PMTCT services in the RCH clinic, which is available 3 days per week (i.e., Wednesday, Thursday and Friday). Approximately 1000 pregnant women receive PMTCT services at the RCH clinic annually [9].

### Study population and sampling

The study included all pregnant women attending the RCH clinic for PMTCT services at KCMC private referral hospital in Moshi municipality. A single population proportion sample size formula was used to calculate the sample size. Based on a study conducted among pregnant women in Dar es Salaam by Price et al. (2016), which reported 8% dissatisfaction with PMTCT services [3], was taken to calculate the sample size. The desired level of Confidence Interval (CI) at 95%, a margin error of 5%, and non-response rate of 10% were included in the formula as follows: N = Z^2^ x P x (100-P)/E^2^ whereby N = Estimated Sample Size, Z = Standard Normal Deviation of 1.96^2^ corresponding to 95% CI, P = Proportion of outcome under study, and E = Marginal Error at 5% [10]. The final sample size was derived by the following formula [10]: Final sample size = Effective sample size/ (1- non-response rate anticipated). The minimum sample size was calculated as 112, and the anticipated non-response rate or dropout was 10% derived from a previous study conducted in the same study setting [11]. Then the final sample size = 112/ (1–0.1) = 124.

Based on the daily attendance in the RCH clinic, a systematic sampling approach was used to select participants for the study. A list of clients attending the clinic obtained from the daily register book was used as a sampling frame. The first participant was selected using a simple lottery technique whereby a YES was written on 4 pieces of paper, and one piece was drawn blindly from a box placed at the registry section. The rest of the participants who met the inclusion criteria were obtained using a systematic sampling technique.

PMTCT services at KCMC are provided 3 days per week (i.e. Wednesday, Thursday, and Friday), with an average of 12 clients attending per day, therefore the study needed 41 days to complete data collection. The number of clients who had to be recruited per day = Estimated sample size/Number of days (124/41 = 3 clients). A formula of N/n was employed to obtain the sampling interval as follows: N = the total number of clients attending at RCH clinic per day and, n = the estimated number of clients to be recruited per day [10]. The procedure was repeated 3 days per week to accomplish the estimated sample size. The inclusion criteria were age 18 years or older, those who are willing to provide consent to participate, and able to coherently communicate in Kiswahili-the local language commonly used in the study settings.

#### Study variables

The dependent variable was pregnant women satisfaction with the quality of care and was measured by the difference between the mean perception scores and mean expectation scores for each of the 5 service dimensions. Quality of care was deemed indifferent or sufficient when pregnant women satisfaction is equal or greater than the expected level of service or vice versa. The socio-demographic attributes of pregnant women include age, level of education, residence, marital status, and occupation.

The 5 service dimensions were assessed as follows: Five questions were used to assess the tangible dimension. An example question for expectations was “I expect the clinic will have ARV drugs easily available". The expected response was 1 = strongly disagree; 4 = strongly agree. An example question for perception was " I am satisfied that the clinic has ARV drugs easily available”. The tangibles scale was Cronbach’s alpha coefficients = .87.

Four questions were used to assess the reliability dimension. An example question for expectations was “I expect staff to have good communication and information skills". The expected response was 1 = strongly disagree; 4 = strongly agree. An example question for perception was " I am satisfied that staff have good communication and information skills”. The reliability scale was alpha = .78.

Seven questions were used to assess the responsiveness dimension. An example question for expectations was “I expect staff will spend enough time to attend their clients". The expected response was 1 = strongly disagree; 4 = strongly agree. An example question for perception was " I am satisfied that staff spend enough time to attend their clients”. The responsiveness scale was alpha = .85.

Five questions were used to assess the assurance dimension. An example question for expectations was “I expect the staff will have time to answer clients’ questions”. The expected response was 1 = strongly disagree; 4 = strongly agree. An example question for perception was “I am satisfied that staff have time to answer clients’ questions”. The assurance scale was alpha = .80.

Five questions were used to assess the empathy dimension. An example question for expectations was “I expect staff to pay attention and listen to medical concerns of their clients". The expected response was 1 = strongly disagree; 4 = strongly agree. An example question for perception was " I am satisfied that staff paid attention and listen to medical concerns of their clients”. The empathy scale was alpha = .82. In this study, Cronbach’s alpha coefficients had a reliability index ranging from .78 to .87. All of the reliability alphas were above the cut-off point of =. 60, which is criterion for internal consistency of new scales [12].

### Data collection

In this study, the SERVQUAL questionnaire was adapted to be applicable to measure the service gap for PMTCT services. The questionnaire was divided into 2 parts: the first part collected demographic characteristics of participants and the second part collected information about pregnant women’ expectations and perceptions of PMTCT services.

According to the SERVQUAL scale, perceptions are based on the actual service received in the RCH clinic while expectations are based on past experiences and information received about the PMTCT services. The SERVQUAL items were translated from English to Kiswahili and back-translated firstly by BN. Kiswahili was used because it is the local language commonly used in Tanzania. Two external specialists also verified the final version of the questionnaire to ensure the reliability of the scale.

The SERVQUAL questionnaire comprised 52 (26 pairs of expectations and perceptions questions) questions comparing participants’ expectations to their perceptions in 5 service dimensions. The 5 service dimensions are as follows: (a) tangibles (i.e., the appearance of the physical facilities, equipment, personnel, and communication materials); (b) reliability (i.e., the ability to perform expected services/care dependably and accurately); (c) responsiveness (i.e., the willingness to help patients and prompt care); (d) assurance (i.e., the knowledge and courtesy of providers and their ability to convey trust and confidence), and (e) empathy (i.e., the provision of caring, access, communication, and understanding to individual clients). Before data collection, the tool was piloted in 20 pregnant women attending the RCH clinic for PMTCT services at a regional referral hospital in the Moshi municipality, to check on its structure, clarity of the items, and ease of completion. Results from the pilot testing indicated that the SERVQUAL questions were clear, understandable, and easy to follow, and were incorporated into the final version of the questionnaire. Three trained fourth-year medical students (L J, NO, & J N) collected data from eligible participants after obtaining written informed consent. Data was collected in a private room within the RCH clinic to ensure confidentiality, and acquire independent and accurate responses. No names of participants were used on the questionnaire but coded numbers to ensure anonymity.

Each questionnaire took approximately 30 min to complete. A total of 105 questionnaires were distributed and filled. Participants rated their expectations and perceptions of the quality of care on a 4-point Likert scale as follows: 1 = strongly disagree; 2 = disagree; 3 = agree; and 4 = strongly agree.

### Ethical considerations

Permission to conduct the study was obtained from the Kilimanjaro Christian Medical College ethical committee (No: 901), and permission to collect data was obtained from the Director of Hospital Services of KCMC hospital. The data collection procedure was explained to all participants before data collection on their voluntary participation and the right to withdraw from the study without any consequences.

### Data management

The data was cleaned, coded, and double entered by two authors (L J & N O), into a computer, analyzed using Statistical Package for Social Science (SPSS) version 23.0 (SPSS Inc., Chicago). Descriptive analysis with frequencies and percentages were generated for participants’ socio-demographic characteristics. To assess the relationship between pregnant women mean service gap scores and their demographic profiles a t-test and ANOVA were used.

The scores associated with the perceived quality of PMTCT services in each dimension and the overall perception scores were calculated. Pregnant women perceived quality of care of PMTCT services were measured by the difference between the mean perception scores and mean expectation scores for each item. According to the SERVQUAL scale, if the gap scores of the perceptions exceed expectations, then the higher perceived quality of care of PMTCT services. In case the expectation and perception are equal, the quality of care of PMTCT services was considered satisfactory. If the expectation exceeded the perception, the quality of care of PMTCT services was considered less satisfactory.

The disparity between expected variables and perceived variables was calculated, using the formula as follows: service quality (Gap Score) = Perceived mean service score (P)–Expectation mean service scores (E). While the average gap score of each dimension was calculated by the average gap score of each dimension = Total means gap score of each dimension / Total number of each dimension’s items (questions). Then, the overall score of service quality was calculated by Overall service quality score = Total average mean gap score of all dimensions / 5 (dimensions).

## Results

### Study participant characteristics

One hundred and five questionnaires were completed (response rate of 84.7%). The mean age of participants was 33 years and a standard deviation (SD) of 6.4 years. Of the 105 participants, 53(50.5%) were aged 26–35 years, 49 (46.6%) were married, 61(58%) had 2 children or less, 59(56.2%) had primary level education, 67(63.8%) were employed, and 83(79%) were residing more than 2 kilometres to the clinic Table 1.

**Table 1:** Demographic characteristics of participants (n = 105).

Variable	Frequency	Percentage (%)
**Age in years**		
≤ 25	12	11.4
26–35	53	50.5
≥ 36	40	38.1
Mean (SD)	33	6.4*
**Marital status**		
Single	28	26.7
Married	49	46.6
Others	28	26.7
**Number of children**		
≤ 2	61	58
3–6	43	41
≥ 6	1	1
Mean (SD)	2.56	1.35*
**Education level**		
Primary education level (class 1–7)	59	56.2
Secondary education level	34	32.4
College/university	12	11.4
** Occupation**		
Unemployed	38	36.2
Employed	67	63.8
**Residence**		
<2km to the clinic	22	21
>2km to the clinic	83	79
* Standard deviation (SD)		

The overall service quality gap scores (i.e., the gap between expectations and perceptions) showed a positive gap score (+ 0.31) in all 5 service dimensions Table 2.

**Table 2:** The overall level of pregnant women satisfaction with the quality of PMTCT service with all 5-service dimensions.

Service dimensions	Perception mean score (SE)	Expectation mean score (SE)	Overall gape score (Service Quality/satisfaction)
Empathy	3.54 (.043)	3.05 (.032)	+0.49
Tangible	3.52 (.031)	3.09 (.017)	+0.43
Assurance	3.41 (.042)	3.20 (.038)	+0.22
Responsiveness	3.25 (.045)	3.05 (.019)	+0.20
Reliability	3.33 (.026)	3.14 (.056)	+0.19
Total mean score	3.41 (.055)	3.10 (.028)	+0.31

Table 3 below summarizes the mean scores and ranking of the 5 service dimensions to assess the quality of care. Of all the 5 service dimensions, empathy was ranked first with the highest gap score (mean gap score = 0.49). The means for all 5 items in the expectations score ranged from the highest 3.13 to the lowest 2.97.

**Table 3:** Mean score and ranking of 5 service dimensions to assess the quality of care (n = 105).

SN	SERVIQUAL statements	Mean perception score (SE)	Mean expectation score (SE)	Mean gap score	P-value
**I**	**Tangibles dimension**				
1	PMTCT clinic provided me with ARV drugs	3.51(.054)	3.08 (.071)	0.43	0.000
2	Doctors of this PMTCT clinic have prescribed good drugs	3.45(.057)	3.06 (.074	0.39	0.000
3	ARV is obtained easily in this PMTCT clinic	3.62(.049)	3.11(.071)	0.41	0.000
4	PMTCT clinic has a good reception area with sufficient seats and toilets	3.47(.052)	3.15(.056)	0.44	0.000
5	PMTCT clinic appears clean every day	3.56(.049)	3.06(.070)	0.44	0.000
	**Average Tangibles SERVIQUAL scores**	**3.52(.031)**	**3.09(.017)**	**0.43**	
**II**	**Reliability dimension**				
6	PMTCT staff keep appointments given to me	3.32(.047)	3.30(.060)	0.02	0.000
7	PMTCT staff have good communication and information skills	3.26(.052)	3.05(.077)	0.21	0.000
8	PMTCT staff has fulfilled my expectations by giving me thorough physical examinations	3.36(.050)	3.08(.063)	0.28	0.000
9	PMTCT staff has given me proper medications as prescribed	3.38(.051)	3.13(.058)	0.25	0.000
	**Average Reliability SERVIQUAL scores**	**3.33 (.026)**	**3.14 (.056)**	**0.19**	
**III**	**Responsiveness dimension**				
10	PMTCT staff retrieve my records promptly	3.35(.049)	3.08(.054)	0.27	0.000
11	PMTCT staff identifies very ill patients and assist them whenever there is a need	3.34 (.026)	3.09 (.059)	0.25	0.000
12	PMTCT staff is respectful to me	3.29(.055)	3.06(.060)	0.23	0.000
13	PMTCT staff offer prompt services	3.12(.067)	3.05 (.073)	0.07	0.000
14	PMTCT staff is willing to help clients whenever medical help is needed	3.36(.054)	3.10 (.061)	0.26	0.000
15	I used a short period to wait(< 30 min) before getting services	3.06(.070)	2.95 (.059)	0.11	0.000
16	PMTCT staff spend enough time (at least 10 min) while attending my problems	3.29(.054)	3.03 (.060)	0.26	0.000
	**Average Responsiveness SERVIQUAL scores**	**3.25 (.045)**	**3.05 (.019)**	**0.20**	
**IV**	**Assurance dimension**				
17	Laboratory results of this PMTCT clinic are timely availed	3.45(.056)	3.25(.064)	0.20	0.000
18	PMTCT staff adhere to the confidentiality of my information	3.44(.062)	3.15(.074)	0.29	0.000
19	PMTCT clinic has adequate staff to take care of the clients	3.26(.062)	3.08(.066)	0.18	0.000
20	PMTCT staff has enough knowledge to answer my questions	3.40(.052)	3.25(.068)	0.15	0.000
21	I will recommend these PMTCT services to other clients	3.51(.054)	3.28(.067)	0.23	0.000
	**Average Assurance SERVIQUAL scores**	**3.41 (.042)**	**3.20 (.038)**	**0.21**	
**V**	**Empathy dimension**				
22	PMTCT staff paid attention to my medical concerns	3.47(.042)	2.97(.067)	0.50	0.000
23	PMTCT staff has built good cooperation with me and are ready to offer me medical assistance.	3.59(.051)	3.03(.066)	0.53	0.000
24	PMTCT staff is polite, comforting and encouraging to me when faced with medical problems	3.56(.054)	3.13(.073)	0.43	0.000
25	PMTCT staff were compassionate to me	3.65 (.055)	3.12(.074)	0.53	0.000
26	PMTCT staff listened to me adequately	3.41 (.042)	3.00 (.061)	0.41	0.000
	**Average Empathy SERVIQUAL scores**	**3.54 (.043)**	**3.05 (.032)**	**0.49**	

The means for all 5 items in the perception score ranged from the highest 3.65 to the lowest 3.41. The largest service gap score was 0.53 for *“PMTCT staff has built good cooperation with me and are ready to offer me medical problems*, and *“PMTCT staff were compassionate to me”*, respectively. The smallest service gap score was 0.41 for the *“PMTCT staff listened to me adequately”* item.

Tangibles were ranked second (mean gap score = 0.43). The means for all 5 items in the expectations score ranged from the highest = 3.15 to the lowest = 3.06. The means for all 5 items in the perception score ranged from the highest = 3.62 to the lowest = 3.45. The largest service gap score was = 0.44 for *“PMTCT clinic appears clean every day”*, and *" PMTCT clinic has a good reception area with sufficient seats and toilets"*, respectively. The smallest service gap score was = 0.39 for the *“Doctors of this PMTCT clinic have prescribed good drugs”* item.

Assurance was ranked third (mean gap score = 0.21). The means for all 5 items in the expectations score ranged from the highest = 3.28 to the lowest = 3.08. The means for all 5 items in the perception score ranged from the highest = 3.51 to the lowest = 3.26. The largest service gap score was = 0.29 for the *" PMTCT staff adhere to the confidentiality of my information”* item. The smallest service gap score was = 0.15 for the *“PMTCT staff has enough knowledge to answer my questions”* item.

Responsiveness was ranked fourth (mean gap score = 0.20). The means for the 7 items in the expectations score ranged from the highest = 3.10 to the lowest = 2.95. The means for the 7 items in the perceptions score ranged from the highest = 3.36 to the lowest = 3.06. The largest service gap score was = 0.27 for *the " PMTCT staff retrieve my records promptly”* item. The smallest service gap score was = 0.07 for the *“PMTCT staff offer prompt services”* item.

Reliability was ranked fifth (mean gap score = 0.19). The means for all 4 items in the expectations score ranged from the highest = 3.30 to the lowest = 3.05 The means for all 4 items in the perceptions score ranged from the highest = 3.38 to the lowest = 3.26. The largest service gap score was = 0.28 for the *" PMTCT staff has fulfilled my expectations by giving me thorough physical examinations”* item. The smallest service gap score was = 0.02 for the *“PMTCT staff keep appointments given to me”* item.

Table 4 below presents the relationship between pregnant women service gap scores and their socio-demographic characteristics. A significant association was found between marital status and the overall gap score (p < 0.05). Other demographic characteristics had no significant association with the service gap (p> 0.05).

**Table 4:** Association of pregnant women service gap scores and socio-demographic characteristics (n = 105).

Variables	P mean score	E mean score	Service gap score	P-Value
Age in years 0.25				
≤ 25	3.91	2.86	1.05	
26–35	3.91	3.14	0.77	
≥36	3.92	3.08	0.84	
Marital status 0.05*				
Single	3.92	3.08	0.84	
Married	3.91	3.22	0.69	
Other	3.93	2.85	1.10	
Number of children 0.18				
≤ 2	3.92	2.99	0.93	
3–5	3.91	3.23	0.68	
≥ 6	4.00	2.70	1.30	
Education level 0.51				
Primary level (class 1–7)	3.93	3.12	0.81	
Secondary level (form 1–6)	3.91	3.08	0.83	
Collage/University	3.89	2.93	0.96	
Occupation 0.21				
Unemployed	3.94	3.26	0.68	
Employed	3.91	2.98	0.93	
Residence 0.22				
< 2 km to clinic	3.98	3.08	0.90	
> 2 km to the clinic	3.90	3.08	0.82	


## Discussion

This study aimed to measure the gap between pregnant women’ expectations of PMTCT services and perceptions of the actual PMTCT services and the relationship between their service gap scores and socio-demographic characteristics. The overall perception scores for all the SERVQUAL dimensions (tangibles, reliability, assurance, responsiveness and empathy) were higher than the corresponding expectation scores. The study finding provides evidence of a positive gap score, indicating that the perceived value of the quality of PMTCT services delivered in the RCH clinic at KCMC referral hospital has exceeded the initial expectation for all 5 service dimensions. This finding concurs with the results of studies conducted globally [4, 5, 13–16]. In contrast, some studies have reported pregnant women dissatisfaction with the quality of care provided at RCH clinics [17–19]. The most probable explanation could be because of variations in study settings, study populations, different levels of expectations among patients, or actual lower levels of perceived quality of services provided [11, 17].

The ability of staff to listen adequately included in the empathy dimension had a small service gap score. This finding corroborates with studies in other settings, which reported staff ability to listen adequately to their client’s increase the ability to create trust and confidence between them [5, 13, 20–25].

In this study, the doctor’s ability to prescribe good drugs included in the tangible dimension was related to a small gap score. This observation concurs with studies conducted in different settings, which reported that a clinician’s ability to prescribe good drugs is significantly related to levels of perceived quality of care provided [26–28].

The statement “PMTCT staff offer prompt services” included in the responsiveness dimension, had a small service gap score. This finding is congruent with findings conducted in other settings, in which health care providers’ willingness to help, promptness of care, and perceived respect enhance patient-provider relationships, and improves client’s perceptions of the quality of care [13, 19, 20, 22, 29]. To meet the service expectations of pregnant women it is imperative to address the issues of long waiting time, with more preference to have more time with the staff, frequent check-ups, and shorter intervals between checkups [14, 30, 31].

In this study, the ability of staff to keep appointments given to clients included in the reliability dimension was related to a small service gap score. Existing evidence shows that keeping clients appointment increases more regular consultations and betterment of the quality of care [7, 13, 17, 19, 29, 32]. In contrast, lack of appointment systems and booking of scheduled appointments in advance may delay pregnant women access to PMTCT services [14, 33, 34].

Finally, in this study marital status was significantly associated with the pregnant women overall service gap score. Pregnant women who were married had a small service gap score with the perceived quality of care of PMTCT services. This observation could be explained by the fact that this group reported higher expectations mean score before receiving the actual PMTCT services, or had bad past experiences with the delivery of PMTCT care ending in a more critical evaluation of their expectations with services [27, 29].

### Study strength and limitations

The strength of this study is the use of a standardized SERVIQUAL questionnaire used in different settings and has achieved consensus for the variables under consideration, hence content validity can be confidently asserted.

Another strength of this study is the back-translation of the Swahili version of the SERVIQUAL questionnaire into English with nearly a perfect match, and also pre-tested for accuracy of meaning and wording, and administered by well-trained research assistants based on a standardized data collection protocol for data collection.

Further, the study assured participants of both confidentiality of data and anonymity of their identity, reducing fear of retaliation or simple social desirability bias. The above measures aimed to minimize the effect of interviewer bias on the study findings.

The study findings should be interpreted with caution because of the nature of the cross-sectional study design. The potential weaknesses of this study include the inability of the cross-sectional study to identify the causality of the outcome of interest. Since the study only recruited pregnant women who attended the RCH clinic and was unable to reach pregnant women without access to RCH care, selection bias cannot be excluded. Also, selection bias is a possibility because the study excluded pregnant women who were younger than 18 years old because of ethical issues, and also pregnant women with severe complications.

Another limitation of our study is the generalizability of the findings because data were collected in one RCH clinic at a private referral hospital in an urban setting. However, Moshi municipality is similar to most urban settings in terms of socio-demographic characteristics of pregnant women and the quality of care, hence allowing extrapolation of the study findings to comparable settings. Variation of socio-demographic characteristics of pregnant women and the quality of care in rural settings do not allow for the generalization of our results to such settings. Final, this study used the quantitative method alone hence future studies using the SERVIQUAL model should incorporate qualitative methods to get an in-depth interpretation of the results.

### Conclusion

The overall service quality gap scores showed a positive gap score in all 5 service dimensions signify that pregnant women perceptions of PMTCT services provided in the RCH clinic at KCMC were meet. Marital status was associated with the overall pregnant women service gap score with the perceived quality of care with PMTCT services. Pregnant women who were married had a small service gap score compared to single or widowed/separated women. The fact that the overall perception levels are higher than expectations for all 5 service dimensions in this study, should not allow the staff at the RCH clinic at KCMC private referral hospital to become complacent, but make efforts to maintain the quality of PMTCT services.

## Supporting information

S1 Table The highest and lowest mean expectations scores (n = 105). (DOCX)

S2 Table The highest and lowest mean perceptions scores (n = 105). (DOCX)
