# Supplementary material for: Gap analysis between expectations and perceptions of pregnant women attending Prevention of Maternal to Child Transmission of HIV services in a private referral hospital in northern Tanzania: A cross-sectional descriptive study
Source: PLoS One. 2021 Sep 22;16(9):e0257771. doi: 10.1371/journal.pone.0257771 (PMC8457476; doi:10.1371/journal.pone.0257771)
Supplement: S1 Table — (DOCX) [file pone.0257771.s001.docx]

|  | |
| --- | --- |
| **S1 Table. The highest and lowest mean expectation scores (n=105)** |  |
| **Aspects of care (dimension)** | **Mean (SE)** |
| *Highest expectation mean scores* | |
| 1. I expect staff to keep appointments given to their clients (reliability) | 3.30 (.060) |
| 2. I expect to recommend the services to other clients (assurance) | 3.28 (.067) |
| 3. I expect the staff has enough knowledge to answer my questions (assurance). | 3.25 (.068) |
| 4. I expect laboratory results of this PMTCT clinic are timely availed (assurance). | 3.25 (.064) |
| 5. I expect PMTCT staff to adhere to the confidentiality of my information | 3.15 (.074) |
| 6. I expect the staff to be polite, comforting and encouraging to their clients when faced with medical problems (empathy). | 3.13 (.073) |
| 7. I expect the PMTCT staff has given me proper medications as prescribed (reliability). | 3.13 (.058) |
| 8. I expect the PMTCT staff will be compassionate to me (empathy) | 3.12 (.074) |
| 9. I expect the clinic will have ARV drugs easily available (tangibles). | 3.11 (.071) |
| 10. I expect PMTCT staff is willing to help clients whenever medical help is needed  (responsiveness). | 3.10 (.061) |
| *Lowest expectation mean scores (dimension)* | |
| 1. I expect PMTCT will provide me with ARV drugs (tangibles). | 3.08 (.071) |
| 2. I expect doctors of this clinic will prescribe good drugs (tangibles). | 3.06 (.071) |
| 3. I expect PMTCT clinic to appear clean everyday (tangibles). | 3.06 (.070) |
| 4. I expect staff to have good communication and information skills (reliability) | 3.05 (.077) |
| 5. I expect staff to offer prompt services (responsiveness). | 3.05 (.073) |
| 6. I expect staff to built good cooperation with me and am ready to offer me medical assistance (empathy). | 3.03 (.066) |
| 7. I expect staff will spend enough time (at least 10 min) to attend their clients (responsiveness). | 3.03 (.060) |
| 8. I expect staff to listen to me adequately (empathy). | 3.00 (.061) |
| 9. I expect staff to pay attention and listen to medical concerns of their clients (empathy). | 2.97 (.067) |
| 10. I expect to use short period of time in queue before getting services (responsiveness | 2.95 (.059) |
| *cut-off point ≥ mean score 3.10  **cut-off point < mean score 3.10 | |
