# Supplementary material for: Gap analysis between expectations and perceptions of pregnant women attending Prevention of Maternal to Child Transmission of HIV services in a private referral hospital in northern Tanzania: A cross-sectional descriptive study
Source: PLoS One. 2021 Sep 22;16(9):e0257771. doi: 10.1371/journal.pone.0257771 (PMC8457476; doi:10.1371/journal.pone.0257771)
Supplement: S2 Table — (DOCX) [file pone.0257771.s002.docx]

|  | |
| --- | --- |
| **S2 Table. The highest and lowest mean perception scores (n=105).** | |
| **Aspects of care (dimension)** | **Mean (SE)** |
| *Highest perception mean scores** | |
| 1. I am satisfied PMTCT staffs were passionate to me (empathy). | 3.65(.055) |
| 2. I am satisfied that ARV drugs are obtained easily (tangibles). | 3.62 (.049) |
| 3. I am satisfied that the staff have build good cooperation and are ready to offer medical assistance (empathy). | 3.59 (.051) |
| 4. I am satisfied that this clinic appears clean every day (tangibles). | 3.56 (.049) |
| 5. I am satisfied that the staffs are polite, comforting and encouraging to their clients when faced with medical problems (empathy). | 3.56 (.054) |
| 6. I am satisfied PMTCT clinic provided me with ARV drugs (tangibles). | 3.51 (.054) |
| 7. I am satisfied that I will recommend these PMTCT services to other clients ( assurance). | 3.51 (.054) |
| 8. I am satisfied PMTCT clinic has good reception area with sufficient seats and toilets (tangibles). | 3.47 (.052) |
| 9. I am satisfied that PMTCT staff paid attention to my medical concerns (empathy). | 3.47 (.042) |
| 10. I am satisfied that laboratory results of this PMTCT clinic are timely availed (assurance). | 3.45 (.056) |
| *Lowest perception mean scores (dimension)*** | |
| 1. I am satisfied that PMTCT staff has given me proper medications as prescribed (reliability). | 3.38 (.051) |
| 2. I am satisfied that PMTCT staffs are willing to help clients whenever medical help is needed (responsiveness). | 3.36 (.054) |
| 3. I am satisfied that staff retrieves my records promptly (responsiveness) | 3.35 (.049) |
| 4. I am satisfied PMTCT staff identifies very ill patients and assist then whenever there is need (responsiveness). | 3.34 (.026) |
| 5. I am satisfied that keep appointments given to their clients (reliability). | 3.32 (.047) |
| 6. I am satisfied that staff have respect to their clients (responsiveness). | 3.29 (.055) |
| 7. I am satisfied that staff have good communication and information  skills (reliability). | 3.26 (.052) |
| 8. I am satisfied PMTCT Clinic has adequate staffs to take care of clients (assurance ). | 3.26 (.062) |
| 9. I am satisfied PMTCT staff offer prompt services (responsiveness). | 3.12 (.067) |
| 10. I am satisfied I used a short period of time to wait (< 30 min) before getting services (responsiveness). | 3.06 (.070) |
| *cut-off point ≥ mean score 3.41  **cut-off point < mean score 3.41 | |
